# Supplementary material for: Tumoricidal Activity of Simvastatin in Synergy with RhoA Inactivation in Antimigration of Clear Cell Renal Cell Carcinoma Cells
Source: Int J Mol Sci. 2023 Jun 4;24(11):9738. doi: 10.3390/ijms24119738 (PMC10253741; doi:10.3390/ijms24119738)
Supplement: Supplementary file 1 [file ijms-24-09738-s001.zip › Figure S1. raw data8.pdf]

Fig. 2C_1

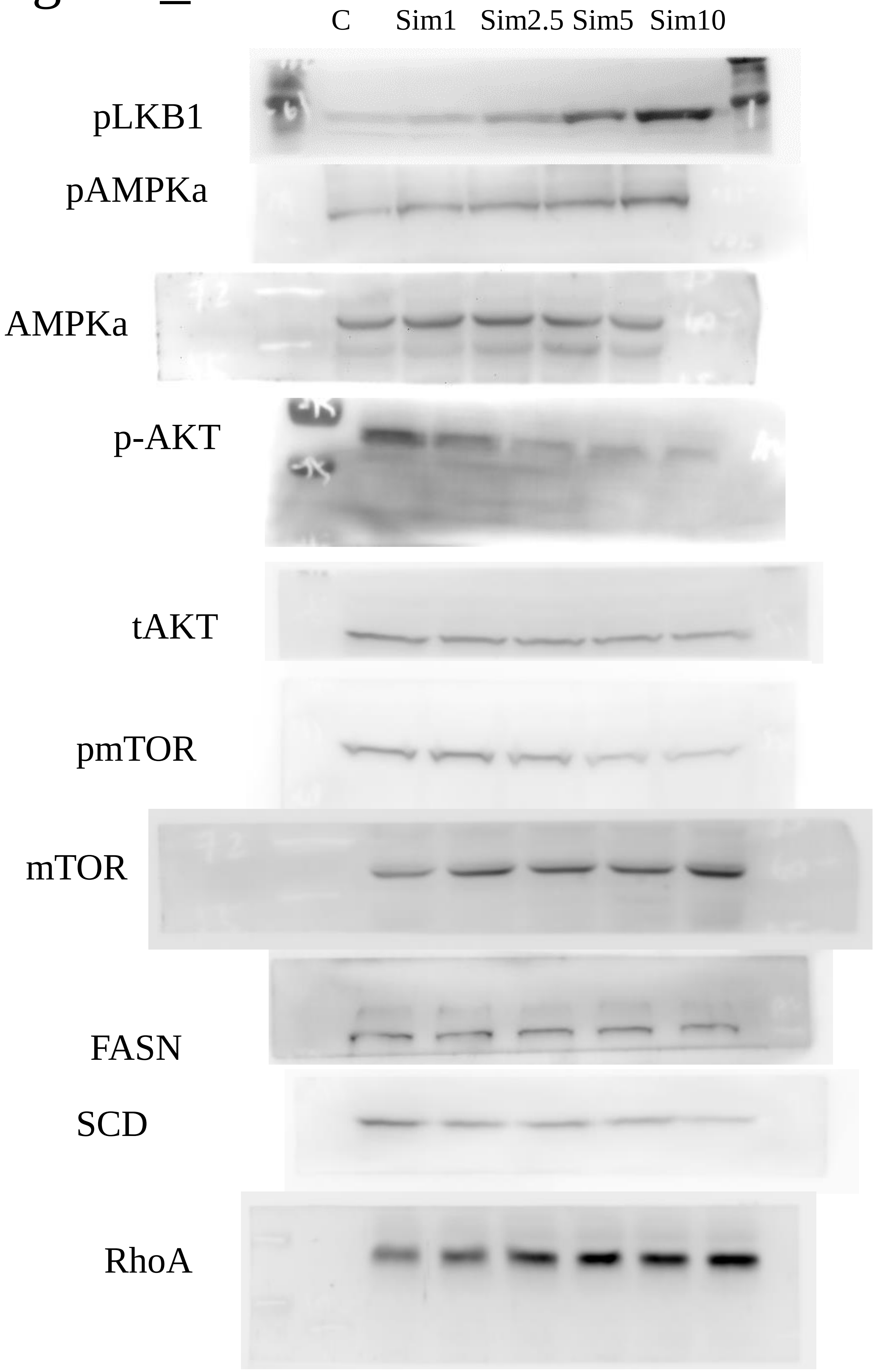
C
Sim1
Sim2.5
Sim5
Sim10
pLKB1
pAMPKa
AMPKa
p-AKT
tAKT
pmTOR
mTOR
FASN
SCD
RhoA

Fig. 2C_2

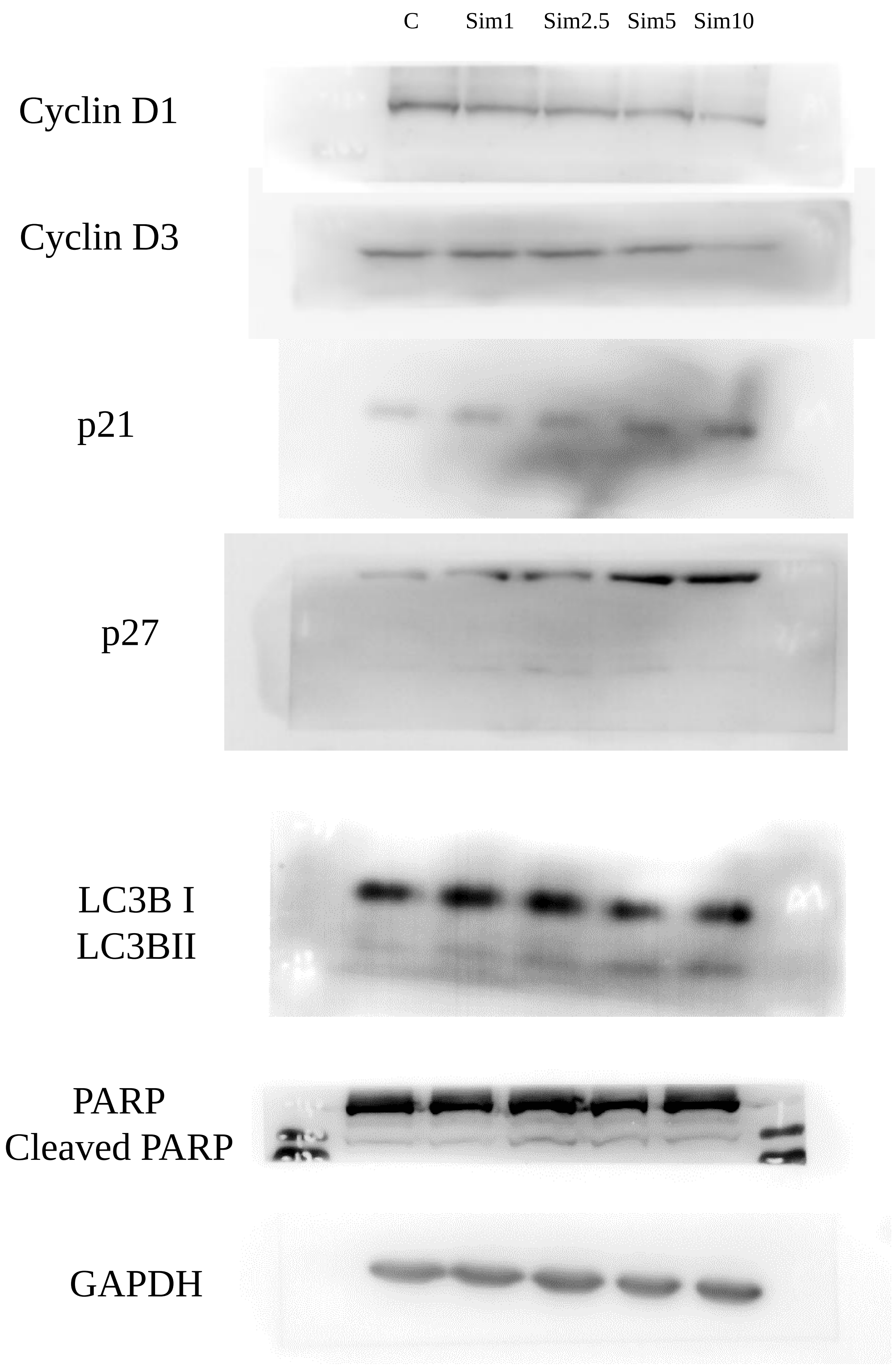
C
Sim1
Sim2.5
Sim5
Sim10
Cyclin D1
Cyclin D3
p21
p27
LC3B I
LC3BII
PARP
Cleaved PARP
GAPDH

Fig. 3A_1

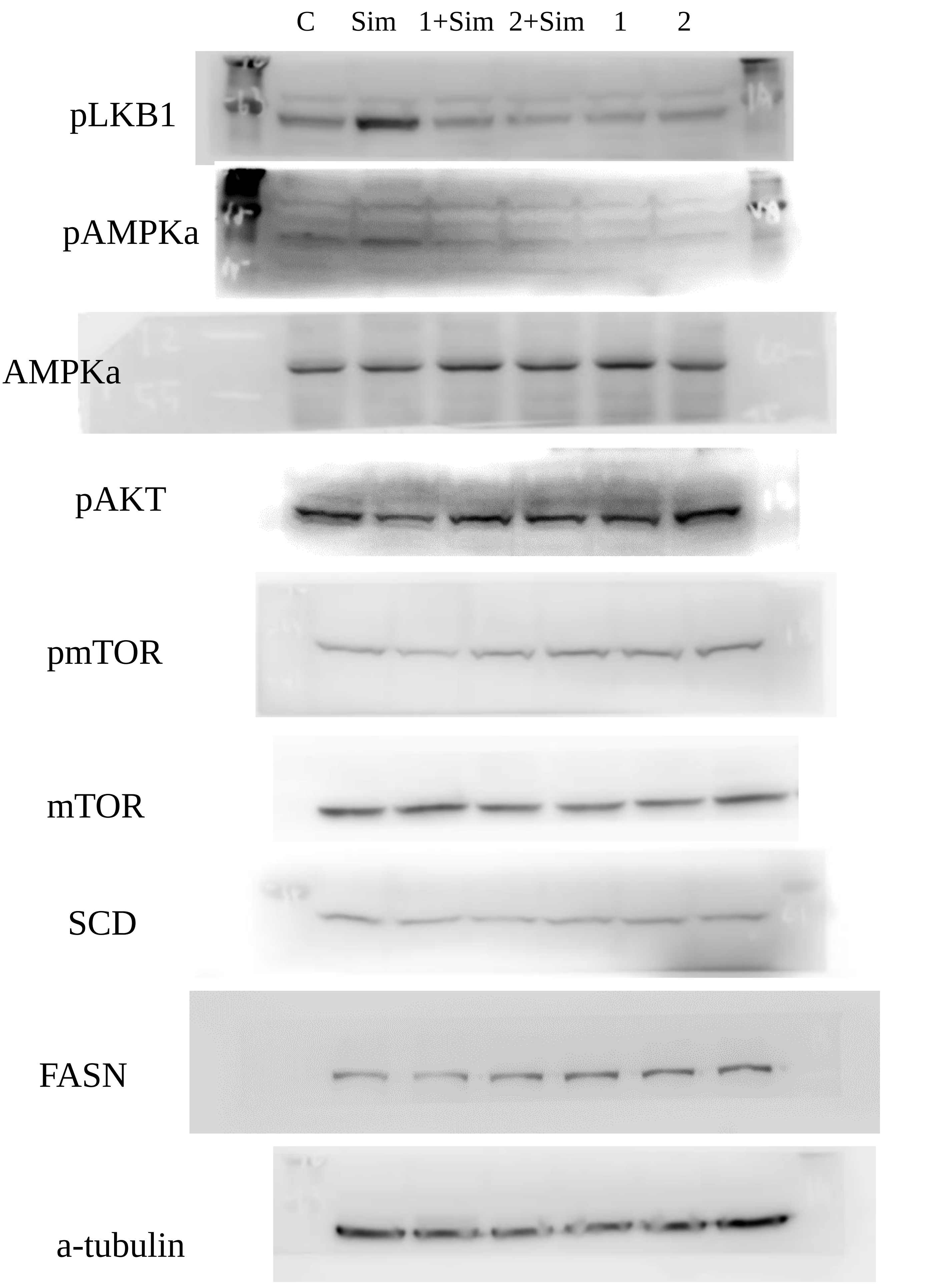
1 : D,L-Mevalonic 0.2 mM
2 : D,L-Mevalonic 0.5 mM
C
Sim
1+Sim
2+Sim
1
2
pLKB1
pAMPKa
AMPKa
pAKT
pmTOR
mTOR
SCD
FASN
a-tubulin

Fig. 3A_2

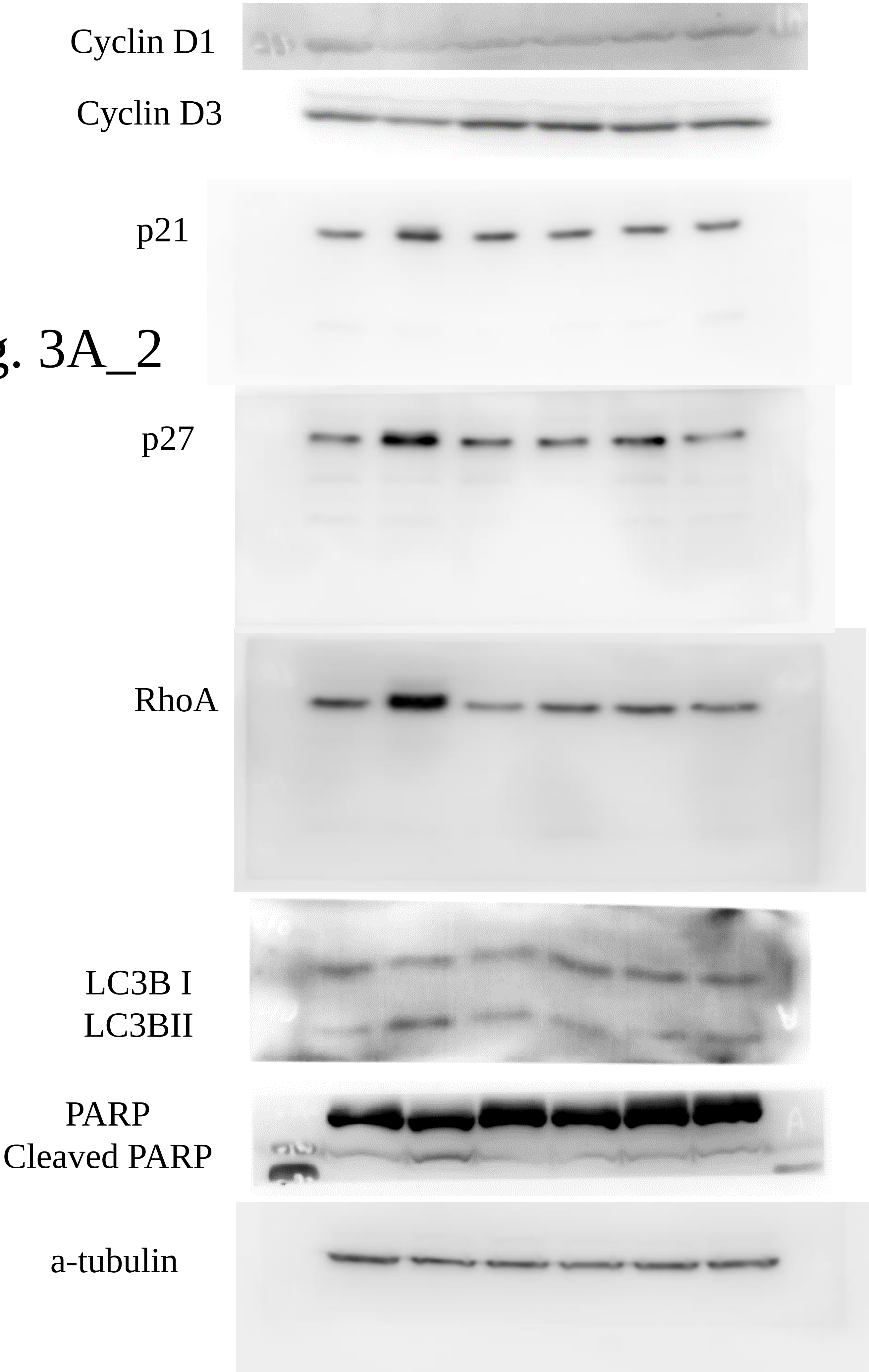

Cyclin D1
Cyclin D3
p21
p27
RhoA
LC3B I
LC3BII
PARP
Cleaved PARP
a-tubulin

Fig. 5A

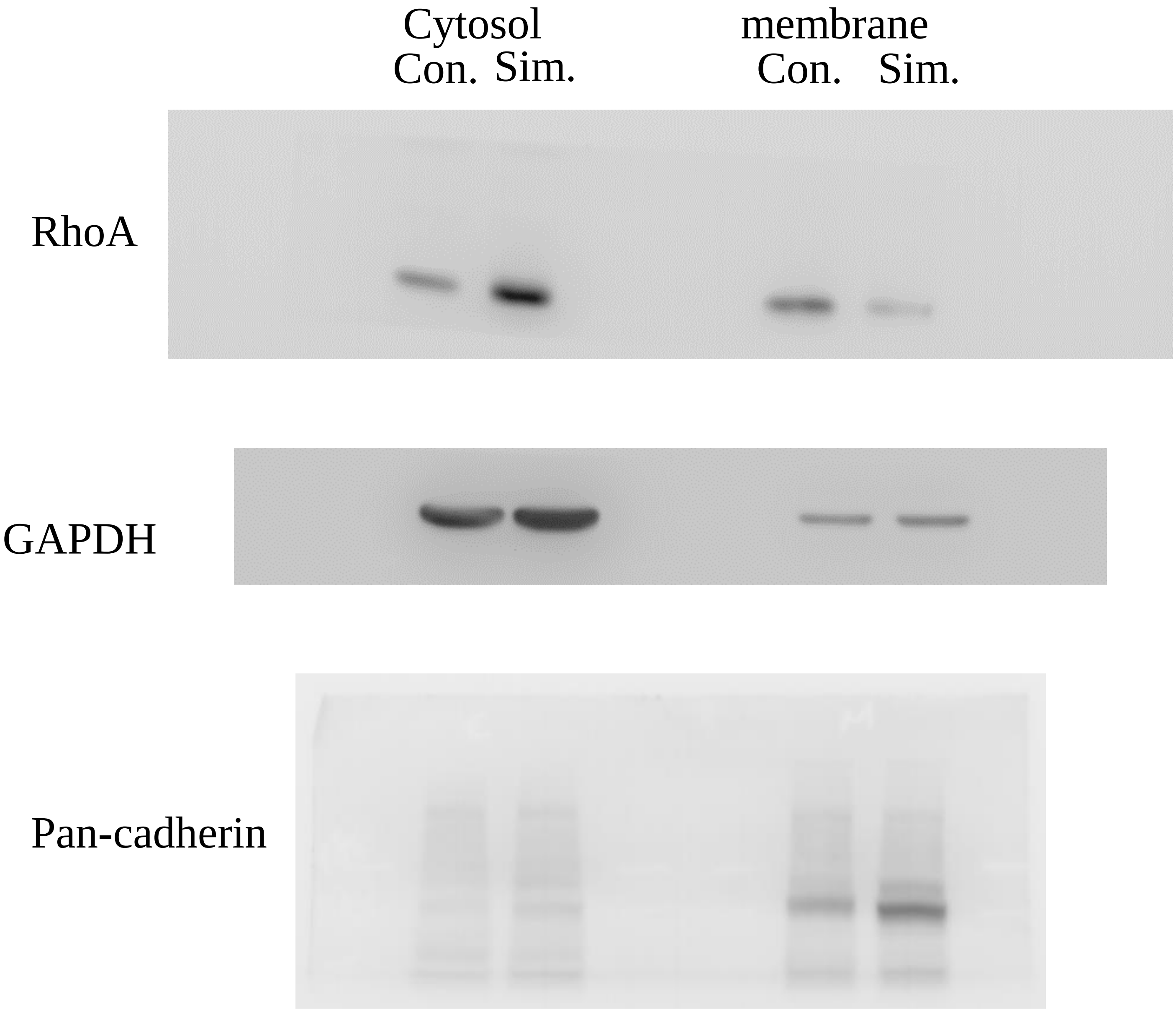

Fig. 5B

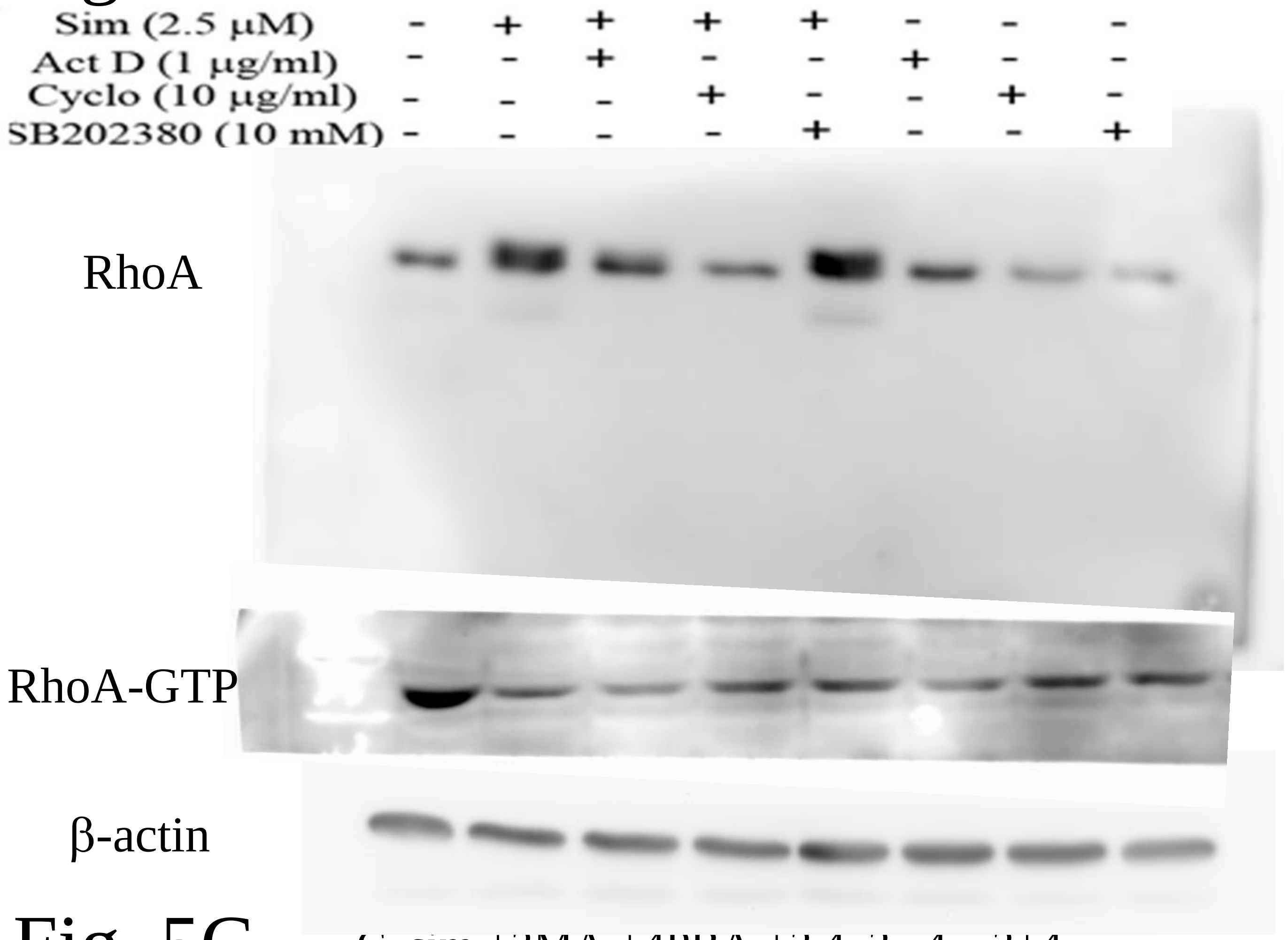
Sim (2.5 µM) - + + + + - - -
Act D (1 µg/ml) - - + - - + - -
Cyclo (10 µg/ml) - - - + - - + -
SB202380 (10 mM) - - - - + - - +
RhoA
RhoA-GTP
β-actin

Fig. 5C

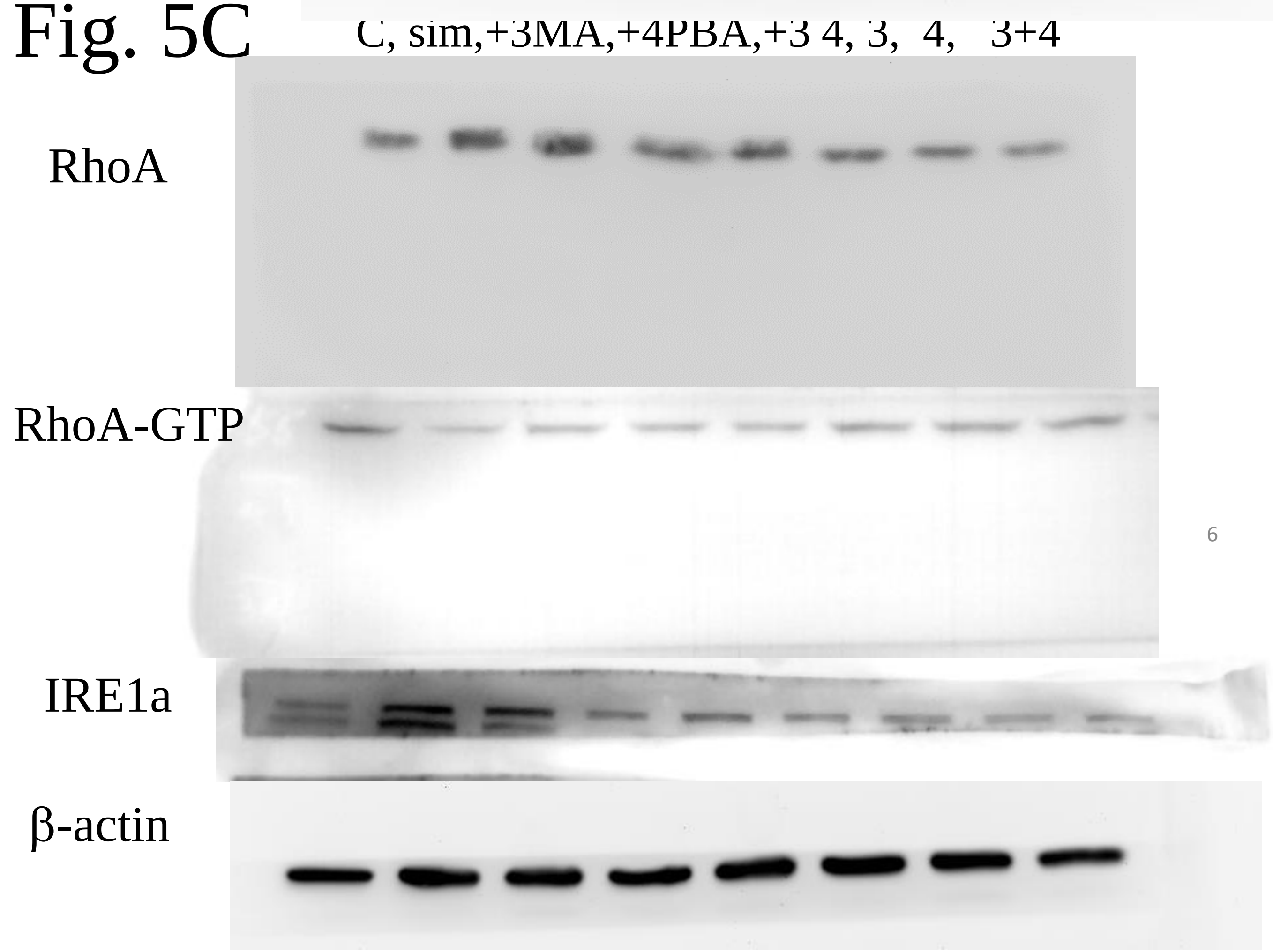
C, sim,+3MA,+4PBA,+3 4, 3, 4, 3+4
RhoA
RhoA-GTP
IRE1a
β-actin

Fig. 6D_1

Vector Ctl DN-, CA-RhoA

pAMPKa

pAKT

MMP2

p4EBP1

LC3BI

LC3BII

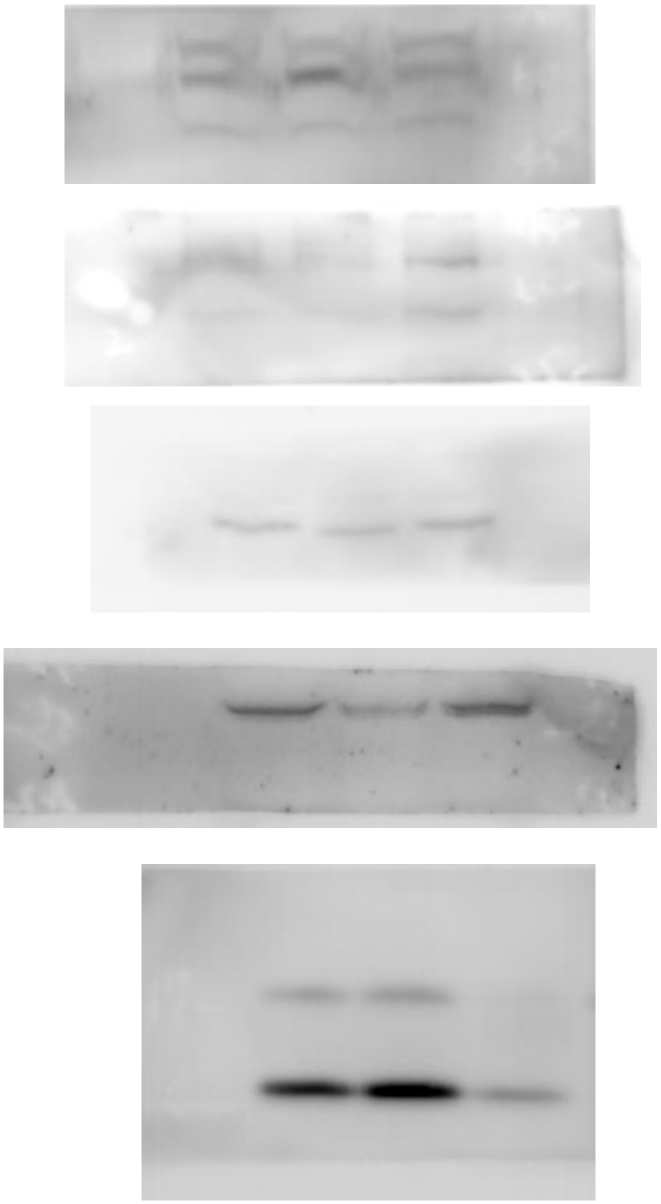

Fig. 6D_2

Vector Ctl DN-, CA-RhoA

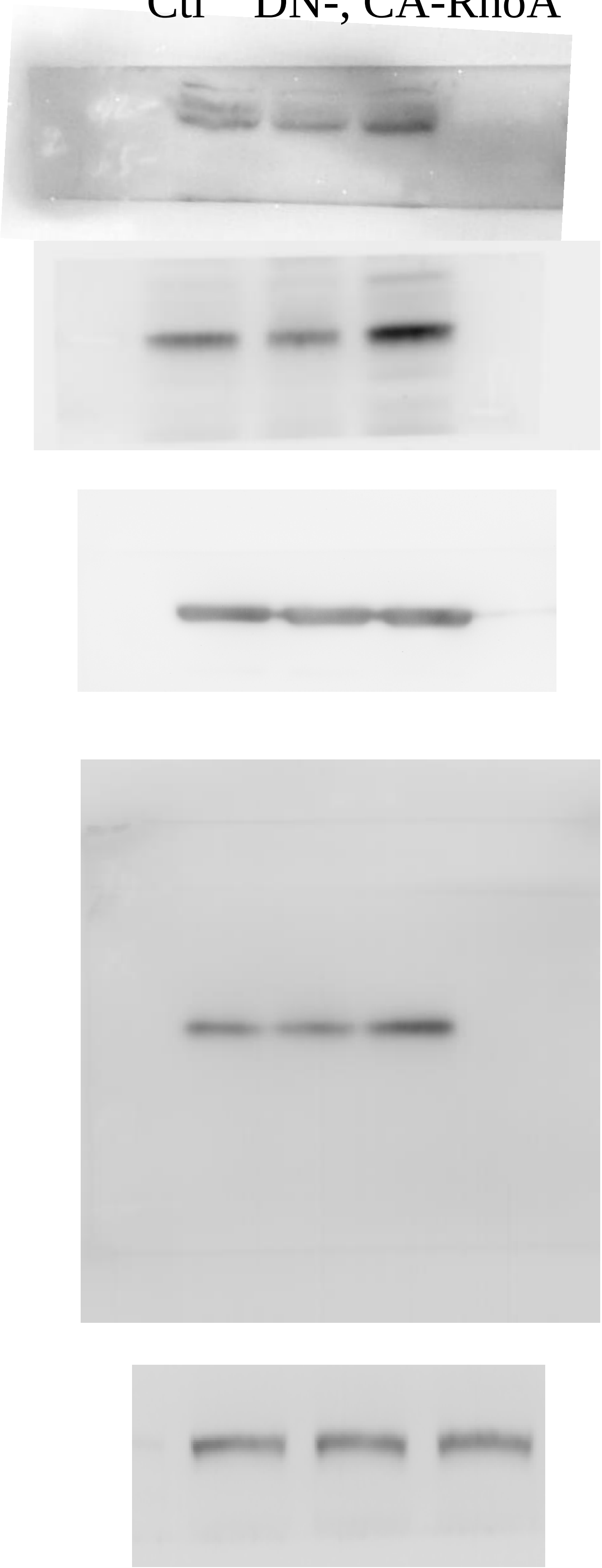

Limk2

p-MLC

β-Actin

RhoA

Pan-cadherin
